# Anorexigenic Effects of Intermittent Hypoxia on the Gut—Brain Axis in Sleep Apnea Syndrome

**DOI:** 10.3390/ijms23010364

**Published:** 2021-12-29

**Authors:** Ryogo Shobatake, Hiroyo Ota, Nobuyuki Takahashi, Satoshi Ueno, Kazuma Sugie, Shin Takasawa

**Affiliations:** 1 Department of Neurology, Nara Medical University, 840 Shijo-cho, Kashihara 634-8522, Japan; sueno@naramed-u.ac.jp (S.U.); ksugie@naramed-u.ac.jp (K.S.); 2 Department of Neurology, Nara City Hospital, 1-50-1 Higashikidera-cho, Nara 630-8305, Japan; takahashin@nara-jadecom.jp; 3 Department of Biochemistry, Nara Medical University, 840 Shijo-cho, Kashihara 634-8521, Japan; shintksw@naramed-u.ac.jp; 4 Department Respiratory Medicine, Nara Medical University, 840 Shijo-cho, Kashihara 634-8522, Japan; hiroyon@naramed-u.ac.jp

**Keywords:** sleep apnea syndrome, intermittent hypoxia, appetite, neuronal cells, enteroendocrine cells, proopiomelanocortin, cocaine- and amphetamine-regulated transcript, peptide YY, glucagon-like peptide-1, neurotensin

## Abstract

Sleep apnea syndrome (SAS) is a breathing disorder characterized by recurrent episodes of upper-airway collapse, resulting in intermittent hypoxia (IH) during sleep. Experimental studies with animals and cellular models have indicated that IH leads to attenuation of glucose-induced insulin secretion from pancreatic β cells and to enhancement of insulin resistance in peripheral tissues and cells, such as the liver (hepatocytes), adipose tissue (adipocytes), and skeletal muscles (myocytes), both of which could lead to obesity. Although obesity is widely recognized as a major factor in SAS, it is controversial whether the development of SAS could contribute directly to obesity, and the effect of IH on the expression of appetite regulatory genes remains elusive. Appetite is regulated appropriately by both the hypothalamus and the gut as a gut–brain axis driven by differential neural and hormonal signals. In this review, we summarized the recent epidemiological findings on the relationship between SAS and feeding behavior and focused on the anorexigenic effects of IH on the gut–brain axis by the IH-induced up-regulation of proopiomelanocortin and cocaine- and amphetamine-regulated transcript in neuronal cells and the IH-induced up-regulation of peptide YY, glucagon-like peptide-1 and neurotensin in enteroendocrine cells and their molecular mechanisms.

## 1. Introduction

Sleep apnea is a sleep disorder in which pauses in breathing or periods of shallow breathing during sleep occur more often than normal. Each pause can last for a few seconds to a few minutes and they happen many times a night. Sleep apnea may be either obstructive sleep apnea (OSA), in which breathing is interrupted by a blockage of air flow, central sleep apnea (CSA), in which regular unconscious breath simply stops, or a combination of the two [1]. Sleep apnea syndrome (SAS), which includes OSA, CSA, and the combination of the two, is one of the common forms of sleep disorder, characterized by repetitive episodes of oxygen desaturation during sleep, the development of daytime sleepiness, and a reduction in quality of life [2]. SAS is associated with many systemic complications, such as obesity; type 2 diabetes mellitus (DM) [3,4,5,6]; dyslipidemia [7]; cardiovascular disease, including hypertension, coronary disease, heart failure, and stroke [8]; pulmonary hypertension [9]; neurocognitive deficits [10,11,12]; and depression [13].

SAS is often caused by a partial or total closure of the upper airway, resulting in a reduction in airflow during sleep, and the prevalence of SAS defined by an apnea–hypopnea index (AHI) ≥ 5 was a mean of 22% in men and 17% in women in eleven published epidemiological studies published between 1993 and 2013 [14]. During sleep, repeated episodes of apnea and/or hypopnea in SAS patients, whether the type is OSA, CSA, or the combination of the two, can result in organs’ and tissues’ exposure to the alternation of low oxygen pressure and normal oxygen pressure, that is, intermittent hypoxia (IH) [6]. IH generates oxidative stress abnormalities that are similar to those seen in ischemia-reperfusion injury [15,16,17,18,19] and lead to redox-activated signal transduction pathways in inflammation [20,21,22]. IH has also been extensively reported to induce sympathetic excitation, leading to IH-induced cardiovascular complications [23]. Many authors have described a variety of causes among SAS patients, although obesity is a major risk factor. Other than obesity, many factors, including tonsillar hypertrophy, macroglossia, narrowed nasal cavities, laxity of the soft palate, and retrognathia, have been involved in the pathogenesis of SAS as anatomical factors [24]. As non-anatomical pathophysiological factors, instability of ventilatory control, also known as high loop gain; neuromuscular inefficiency of the dilator muscles of the upper airways; and an increased inclination for nocturnal awakenings due to respiratory stimuli or a reduced awakening threshold, also known as low arousal threshold, have been reported [25,26].

In this review, we gave an overview of the current knowledge on IH, a hallmark manifestation of SAS, and highlighted the findings of our own cellular studies on the effect of IH on glucose metabolism and the expression of appetite-regulating genes.

## 2. The Reciprocal Relationship between IH and Obesity

Obesity is a very common challenge in association with the metabolic syndrome, the commonly used term for the cluster of obesity, insulin resistance, hypertension, and dyslipidemia [27]. Obesity can cause SAS due to the narrowing of airways induced by an excess of fat tissue around the neck, which can predispose a patient to airway obstruction [28]. In fact, obesity is one of the most important risk factors for the development of SAS [29,30,31], and more than 70% of patients with SAS are obese [32]. Olga et al. have also shown that SAS is considerably present in severely obese patients [33]. A longitudinal study by Peppard and his colleagues indicated that a 10% gain in body weight increased the odds of developing moderate or severe SAS by six-fold [34].

On the other hand, it is reported that about 20% of the adult population with SAS are not obese [35]. A prospective non-randomized controlled study revealed that body mass index (BMI) was significantly lower in SAS Far East-Asian men than that in SAS white men when controlled for sex, age, and disease severity, and that the mean BMI of the Far East-Asian men with SAS was below the norms for men in the United States [36]. A community study in China also showed that BMI < 25 was an independent risk factor for SAS [37]. Thus, it is controversial whether the development of SAS directly contributes to obesity, although a previous systematic review concluded that the energy balance in SAS patients appeared altered to have a positive energy balance [38].

Although the etiology of overweight and obesity is complex, and energy balance is regulated by many neurobiological and physiological mechanisms, weight gain generally results from excessive food intake driven by excessive appetite, which leads to a positive energy balance. Accumulating evidence indicates that obesity and SAS are strongly related to each other [39]; however, the associations between dietary habits or amount and the predisposition to SAS are not fully understood. Regarding food preference, it was reported that the severity of SAS is associated with a liking for high-fat food, based on the respiratory disturbance index in a hierarchical multiple regression model including sex and BMI [40]. A cross-sectional study of 243 patients (21–70 years old) diagnosed with SAS by overnight attended polysomnography in Greece were recruited and showed a positive association between total red meat or unprocessed red meat intake and apnea or hypopnea indices [41]. Another cross-sectional study of 269 patients (21–70 years old, 73.2% males) diagnosed with SAS via an attended in-hospital polysomnography were recruited and reported that SAS is associated with cereal grain intake and suggested that a higher intake of refined cereal grains may be a risk factor for SAS severity [42]. A clinical study with 5076 Mexican adults (20–59 years old) showed that the industrialized dietary pattern (high in sugar-sweetened beverages; fast foods; and alcohol, coffee, or tea) yielded higher odds of sleep apnea (odds ratio 1.63) compared with the traditional dietary pattern (high in legumes and tortillas) [43]. Accordingly, there are various trends in the food preferences of patients with SAS. Moreover, regarding the dietary amount in SAS patients, few studies to date have investigated actual measures of food intake, although one study demonstrated that higher amounts of food intake during the evening period may diminish sleep quality in moderate and severe SAS patients [44]. It is still undetermined whether SAS patients’ appetite is increased or not in spite of several recent epidemiological studies.

## 3. Molecular Mechanisms of the IH-Induced Gene Expression

Many studies have investigated the effects of IH on animals, individuals, and tissues; however, the molecular mechanisms of IH-regulated gene expression are not fully understood. As one of the major mediators associated with IH, much attention has been paid to hypoxia-inducible factor (HIF)-1. IH vigorously activates HIF-1 [45,46,47,48], a transcriptional activator that plays an essential role in regulating cellular adaptive mechanisms in response to a low-oxygen environment [49,50]. Three members of the HIF family have been identified, all of which consist of a heterodimeric structure composed of an O_2_-sensitive α subunit (HIF-1α, HIF-2α, and HIF-3α) and an O_2_-insensitive β subunit (HIF-1β), also known as aryl hydrocarbon receptor nuclear translocator (ARNT) [51]. HIF-1α is responsible for transcriptional activity, as it includes the transactivation domains, and its stability and functionality are controlled by the cellular oxygen pressure [52]. In fact, HIF-1α serum protein concentration was higher in patients with SAS compared with control patients in both the evening (1490.1 pg/mL vs. 727.0 pg/mL; *p* < 0.001) and the morning (1368.9 pg/mL vs. 702.1 pg/mL; *p* < 0.001) samples [53].

Cell cultures and animals exposed to IH have shown HIF-1–dependent transcriptional activation of NADPH oxidases (Noxes) [54,55,56], leading to the enhancement of reactive oxygen species (ROS) production [56]. In turn, ROS generation is essential for increased HIF-1α expression in response to IH [46]. Regarding IH-regulated gene expression, it has been reported that lysine demethylases (KDMs) facilitate HIF-1-dependent transcriptional activation of certain genes by hypoxia [57]. Furthermore, it was reported that under IH, KDM6B is recruited to hypoxia-response element (HRE) binding sites through interaction with HIF-1α and that KDM6B regulates HIF-1 transcriptional activity by demethylating H3K27, of which methylation is regulated by increased KDM6 enzyme activity [58]. Thus, there is increasing evidence of possible IH-induced gene regulatory mechanisms, including epigenetic regulation.

## 4. IH and Impaired Glucose Tolerance

SAS is an independent risk factor for the development and progression of type 2 DM [59] and for insulin resistance [60]. The retrospective study also indicated that desaturation parameters assessed by polysomnography examination are associated with an increased risk of type 2 DM [61]. The level of serum HIF-1α was found to be significantly increased in patients with type 2 DM compared to a control group [62], and cell culture studies show that HIF-1α regulates both glucose uptake and glycolytic enzyme activity, significantly promoting the process of glycolysis [63].

IH is reported to cause β cell replication and apoptosis without hyperglycemia [64], suggesting a possible mechanism by which IH acts as a β cell replication factor. In fact, Ota et al. have demonstrated that IH significantly decreases the gene expression of cluster of differentiation (CD)38 (ADP-ribosyl cyclase/cyclic ADP-ribose [cADPR] hydrolase: EC 3.2.2.6) [6], which is essential for glucose-induced insulin secretion through the mobilization of Ca^2+^ from the intracellular Ca^2+^ pool via type 2 ryanodine receptor Ca^2+^ channel, by cADPR in primary cultured rat and mouse pancreatic islets and animal model experiments [65,66,67,68,69,70,71]. IH also increased rodent pancreatic β cell replication by up-regulation of the regenerating gene (*Reg*) family of genes, which encode autocrine and paracrine growth factors for β cell replication [72,73,74,75], and by the up-regulation of an antiapoptotic hepatocyte growth factor, the up-regulation of which may combat the occurrence of β cell dysfunction and insulin resistance [76]. In human hepatocytes, Uchiyama et al. demonstrated that IH stress up-regulates the levels of *SELENOP*, which encodes selenoprotein P, a causative factor of insulin resistance, and up-regulates the levels of *hepatocarcinoma-intestine-pancreas/pancreatitis-associated protein* (*HIP/PAP*) mRNAs to proliferate the hepatocytes, via the microRNA (miR)-203 mediated mechanism, resulting in the proliferation of liver cells with high levels of *SELENOP* mRNA [5]. Concerning miR-203-mediated mechanisms in IH, Takeda et al. recently reported that the expression of renin in juxtaglomerular cells was significantly increased in response to IH stimulation via down-regulation of miR-203 [77]. The most common complications in SAS patients are hypertension and diabetes, and IH (caused by SAS) decreases miR-203 in hepatocytes [5] and juxtaglomerular cells [77], resulting in increased selenoprotein P in hepatocytes (a diabetogenic hepatokine) and renin in juxtaglomerular cells (which induces hypertension) simultaneously. Uchiyama et al. also indicated that the expression of resistin (RETN), tumor necrosis factor-α (TNF-α), and C-C motif chemokine ligand 2 (CCL2), which are bioactive mediators produced and released from adipocytes and called adipokines, was increased by IH via down-regulation of miR-452 [78]. This suggests that the up-regulation of RETN, TNF-α, and CCL2 in SAS patients may induce a pro-inflammatory phenotype of the adipose tissue, leading to the development of insulin resistance and decreased insulin sensitivity, and miR-452 could play crucial roles in the regulation of these gene expressions. Skeletal muscles also play a major role in insulin-sensitive glucose uptake via glucose transporter 4 (solute carrier family 2, facilitated glucose transporter member 4); however, there are few studies that have examined the effect of IH on glucose uptake and metabolism. Recently, IH was shown to up-regulate some myokines, such as IL-8, osteonectin (also known as secreted protein acidic and rich in cysteine), and myonectin (also known as C1q/TNF-related protein 15 or erythroferrone), which are all involved in inflammation and glucose metabolism, via transcriptional activation of the myokine genes in human and mouse muscle cells [79,80,81].

**Figure 1:**
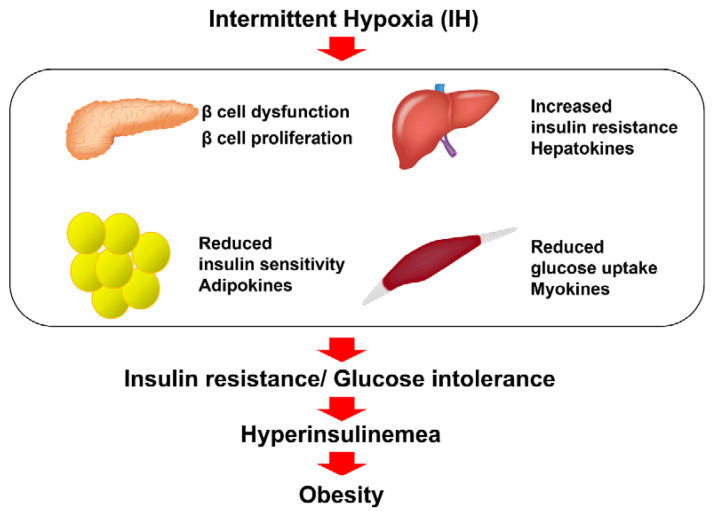
The relationship between IH and insulin resistance, glucose intolerance, and diabetes. IH, frequently observed in SAS patients, is involved in the reduction in glucose-induced insulin secretion from pancreatic β cells via down-regulation of CD38 [6]; up-regulation of Reg I and hepatocyte growth factor in pancreatic β cells [76]; up-regulation of selenoprotein P and HIP/PAP in hepatocytes via down-regulation of miR-203 [5]; up-regulation of adipokines, such as CCL2, TNF-α, and RETN in adipocytes via down-regulation of miR-452 [78]; and up-regulation of myokines, such as IL-8, osteonectin, and myonectin in skeletal muscle cells [79,80,81], all of which can contribute to insulin resistance, glucose intolerance, and obesity [82].

Taken together, there is accumulating evidence indicating that IH induces the impairment of glucose tolerance and insulin resistance in pancreatic β cells, hepatocytes, adipocytes, and skeletal muscle cells, which may contribute to obesity [82] (Figure 1).

## 5. Appetite Regulation in SAS Patients

In appetite regulation, hypothalamic neuroendocrine cells control homeostasis through the production and secretion of neurohormones into the systemic circulation [83]. The hypothalamus is a crucial relay region for integrating signals from central and peripheral pathways in the neuronal circuits controlling energy homeostasis [84,85], being constituted of distinct hypothalamic nuclei, including the arcuate nucleus (ARC), the paraventricular nucleus, the lateral hypothalamic area, the dorsomedial nucleus, and the ventromedial nucleus. In particular, the ARC is considered one of the best characterized areas of the brain involved in the regulation of feeding behavior through the close coordination of multiple neuronal populations [86]. The ARC contains two main neuronal populations with opposite effects on the feeding behavior: the neurons that express orexigenic neuropeptide Y/agouti-related peptide (NPY/AGRP) and those that express anorexigenic proopiomelanocortin/cocaine- and amphetamine-regulated transcript (POMC/CART), both of which constitute the central melanocortin system, with downstream target neurons expressing the melanocortin 3 receptor (MC3R) and melanocortin 4 receptor (MC4R) [84]. NPY/AGRP neurons are inhibited by leptin, insulin, and the enteric hormone peptide YY (PYY)_3-36_, and they are stimulated by ghrelin (GHRL), an orexigenic hormone released from gastric mucosa [87]. Galanin (GAL) is an orexigenic neuropeptide expressed by a majority of the noradrenergic neurons in many tissues throughout the body, including the hypothalamus [88]. Pyroglutamylated RFamide peptide (QRFP) is another orexigenic neuropeptide and is produced in cells of the paraventricular and ventromedial nuclei of the hypothalamus in humans [89]. Galanin-like peptide (GALP) is a neuropeptide responsible for energy homeostasis discovered in the porcine hypothalamus. GALP mRNA has also been detected in the human brain, and it has both species- and time-dependent effects on feeding and body weight in rodents [90].

Appetite regulation governed by the central nervous system (CNS) responds to a short-term signal from gastrointestinal (GI) hormones to control food intake and to a long-term signal from adipose tissue to ensure energy storage [91]. In addition to the CNS, the enteric nervous system (ENS) works together as the gut–brain axis, representing a bi-directional signaling axis, along which neurotransmitters contribute to conveying information from the gut to the brain via afferent fibers and sending appropriate signals from the brain via efferent fibers to control gut secretion and motility [92]. The gut–brain axis is driven by differential signals, including neural and hormonal signals, as a key mechanism to transmit information from the gut to the brain via the vagus nerve [93,94,95].

Gut peptides released from enteroendocrine cells lying within the epithelium throughout the GI tract activate vagal and spinal afferents indirectly via activation of neurons of the ENS and relay nutrient-derived energy signals to the brain so that appetite and food intake can be regulated appropriately through the gut–brain axis [96].

The regulation of appetite and food intake requires various circulating peptides and hormones, including hypothalamic factors (POMC, CART, GAL, GALP, orexin, NPY, QRFP, AGRP), gut hormones (GHRL, glucagon-like peptide [GLP-1], PYY, neurotensin [NTS]), and adiposity signals (leptin, insulin). Within these peptides, the hunger signals AGRP, GHRL, orexin, GAL, and NPY stimulate eating behavior, while satiety peptides POMC, CART, QRFP, GLP-1, PYY, NTS, and leptin terminate food consumption.

In SAS, leptin, a satiety signal and a gut hormone, has been the most intensively investigated. Most studies have reported significantly higher levels of serum leptin in SAS than in controls [97,98,99]. However, no significant differences in serum leptin levels have been reported between SAS and control groups [100,101]. A meta-analysis demonstrated that plasma and serum GHRL levels had no significant differences between the SAS group and the control groups [102]. There are no available data on the serum levels of POMC, CART, GAL, GALP, AGRP, QRFP, and NTS in patients with SAS. It was reported that the serum level of fasting GLP-1 was elevated in the SAS group without diabetes [103]. It was reported that the plasma levels of NPY and PYY were similar to those of the controls [99].

## 6. IH in Neuronal Cells

The mechanism by which IH affects SAS patients’ appetite regulation has not been fully elucidated. While many clinical studies have investigated the relationship between appetite control and SAS by means of serum examination, few studies have investigated the gene expressions of the major appetite regulatory genes in IH-treated cells.

We previously investigated the effect of IH on the expression(s) of major appetite regulatory neuropeptide and receptor genes, such as *POMC*, *CART*, *GAL*, *GALP*, *GHRL*, *QRFP*, *AGRP*, *NPY*, and *MC4R* using human neuronal cells (NB-1, SH-SY5Y, and SK-N-SH) and an in vitro IH system, which is a controlled gas delivery system that regulates the flow of nitrogen and oxygen to generate IH, and demonstrated that IH significantly increases the mRNA levels of *POMC* and *CART*, which are anorexigenic neuropeptides, in human neuronal cells. Subsequently, we conducted promotor assays, which indicated that the IH-induced up-regulation of *POMC* and *CART* mRNAs is caused by the transcriptional activation of the *POMC* and *CART* genes and that the −705 to −686 promoter region of the *POMC* gene and the −950 to −929 region of the *CART* gene are essential for the IH-induced promoter activity. Furthermore, using a computer-aided search, we revealed that both the −705 to −686 promoter region of the *POMC* gene and the −950 to −929 region of the *CART* gene contains possible GATA transcription factor binding sequences, and real-time RT-PCR showed that among GATA family members, *GATA2* and *GATA3* mRNAs were mainly expressed in human neuronal cells. Both human *GATA2* and *GATA3* siRNAs were introduced into human neuronal cells, and they abolished the IH-induced up-regulation of *POMC* and *CART* mRNAs, indicating that both GATA2 and GATA3 are key transcription factors for the IH-induced up-regulation of *POMC* and *CART* mRNA expressions [104]. These results suggest that IH can have an anorexigenic effect on patients with SAS through the up-regulation of *POMC* and *CART* mRNA expression via GATA transcription factors in neuronal cells. In terms of clinical research, to date, there are no available data on the serum levels of POMC and CART in patients with SAS. Regarding the significance of GATA factors in IH condition, Park et al. reported the involvement of Gata4 in the IH-induced up-regulation of B cell lymphoma 2 (Bcl-2) and B cell lymphoma-extra large (Bcl-xL) in mouse myocardial cells, although the mechanism by which IH activates Gata4 to induce Bcl-2 and Bcl-xL has been elusive [105].

## 7. IH in Enteroendocrine Cells

Appetite and food intake are controlled not only by the CNS but also by the GI tract, both of which work together as the gut–brain axis. We tested the hypothesis that IH could have an anorexigenic effect on the ENS, as well as the CNS. In addition to neuronal cells, using human and rodent enteroendocrine cell lines (Caco-2 and STC-1) and the same in vitro IH system, we previously investigated the effect of IH on the gene expression (s) of major appetite-inhibiting gut peptide hormones, PYY, GLP-1, and NTS, and explored their gene regulatory mechanism in human enteroendocrine cells exposed to IH. This study showed that IH stress up-regulates the mRNA levels of PYY, GLP-1, and NTS, which are appetite inhibitory hormones, in enteroendocrine cells, suggesting that SAS patients’ appetite could be suppressed in the ENS as well as in the CNS [106]. Regarding the gene regulatory mechanism, the promoter activities of *PYY*, *Glucagon* (*GCG*) (which encodes a preprotein, part of which is cleaved into GLP-1), and *NTS* were not up-regulated by IH. Moreover, real-time RT-PCR showed that the levels of miR-96, miR-527, and miR-2116, which target and silence *PYY*, *GLP-1*, and *NTS*, respectively, in IH-treated cells were not decreased by IH, indicating no involvement of microRNA-mediated posttranscriptional regulation. Subsequently, considering the possibility that the promoter assays did not reflect the authentic chromatin structure of nuclear DNAs (which can alter the transcriptional efficiency), we treated human enteroendocrine cells with 5-azacytidine (5AZC), genistein, trichostatin A (TSA), resveratrol, and quercetin (which affect the epigenetic regulation of gene expression by modifying chromatin structure of nuclear DNAs) and indicated that TSA significantly up-regulated the mRNA levels of *PYY*, *GLP-1*, and *NTS* even in the normoxia condition and that 5AZC significantly decreased the mRNA levels of *PYY*, *GLP-1*, and *NTS* in the IH condition. Furthermore, the combined treatment of TSA and 5AZC recovered the IH-induced up-regulation of *PYY*, *GLP-1*, and *NTS* mRNAs [106]. These results suggest that the IH-induced up-regulation of *PYY*, *GLP-1*, and *NTS* could be caused by an alteration in the chromatin structure of the genes and that TSA has an effect similar to IH and 5AZC has an effect opposite to IH on *PYY*, *GLP-1*, and *NTS* mRNA expressions. In this study, we indicated the possibility that IH has an anorexigenic effect on SAS patients by up-regulating *PYY*, *GLP-1,* and *NTS* gene expressions in enteroendocrine cells and that IH can alter the chromatin structure of the *PYY*, *GLP-1*, and *NTS* genes.

**Figure 2:**
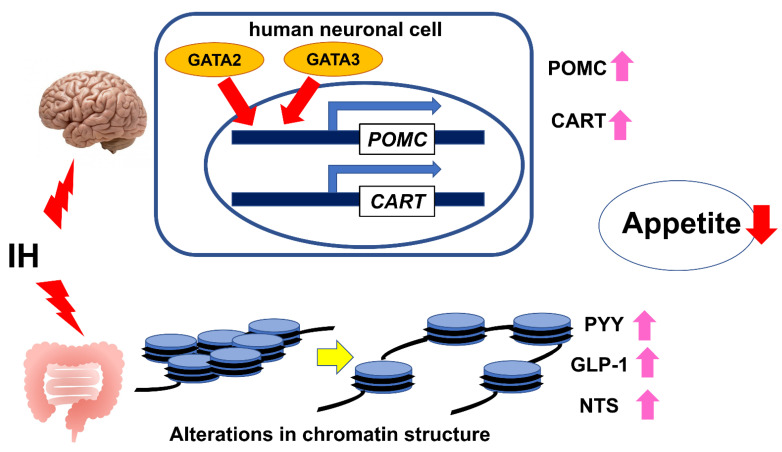
The relationship between IH and the gut–brain axis in appetite control. IH observed in SAS patients can have anorexigenic effects on the gut–brain axis by the up-regulation of *POMC* and *CART* mRNA expression via GATA transcription factors (GATA2 and GATA3) in neuronal cells [104] and by the up-regulation of *PYY*, *GLP-1*, and *NTS* mRNAs through an alteration in chromatin structures of the *PYY*, *GLP-1*, and *NTS* genes in enteroendocrine cells [106].

From these results of our studies on IH-treated neuronal and enteroendocrine cells, IH observed in SAS patients up-regulates the expression of *POMC* and *CART* mRNAs in neuronal cells and *PYY*, *GLP-1*, and *NTS* mRNAs in enteroendocrine cells; therefore, it is possible that IH itself suppresses SAS patients’ appetite through the gut–brain axis (Figure 2).

## 8. Conclusions

Our cellular studies have indicated that IH, a hallmark manifestation of SAS, is involved in the reduction in glucose-induced insulin secretion by down-regulation of CD38 in pancreatic β cells [6]; the up-regulation of selenoprotein P and HIP/PAP via down-regulation of miR-203 in hepatocytes [5]; the up-regulation of adipokines, such as CCL2, TNF-α, and RETN via down-regulation of miR-452 in adipocytes [78]; and the up-regulation of myokines, such as IL-8, osteonectin, and myonectin, in myocytes [79,80,81], all of which can contribute to obesity [82]. On the other hand, we have also revealed the IH-induced up-regulation of *POMC* and *CART* mRNA expression via GATA transcription factors in neuronal cells [104] and the up-regulation of *PYY*, *GLP-1*, and *NTS* mRNAs through alteration in the chromatin structures of the *PYY*, *GLP-1*, and *NTS* genes in enteroendocrine cells [106], both of which suggest possible anorexigenic effects of IH on the gut–brain axis.

To further understand the complex relationship between IH and obesity, more molecular, clinical, and translational research in vitro and in vivo is needed.

## Data Availability

Not applicable.

## Abbreviations

AGRP	Agouti-related peptide
AHI	Apnea-hypopnea index
ARC	Arcuate nucleus
ARNT	Aryl hydrocarbon receptor nuclear translocator
5AZC	5-Azacytidine
Bcl-xL	B cell lymphoma-extra large
Bcl-2	B cell lymphoma 2
BMI	Body Mass Index
CART	Cocaine- and amphetamine-regulated transcript
cADPR	Cyclic ADP-ribose
CCL2	C-C motif chemokine ligand 2
CD38	Cluster of differentiation 38
CNS	Central nervous system
CSA	Central sleep apnea
CXCL2	Chemokine (C-X-C motif) ligand 2
DM	Diabetes mellitus
DNA	Deoxyribonucleic acid
ENS	Enteric nervous system
GAL	Galanin
GALP	Galanin-like peptide
GCG	Glucagon
GLP-1	Glucagon-like peptide-1
GHRL	Ghrelin
GI	Gastrointestinal
HIF	Hypoxia-inducible factor
HIP/PAP	Hepatocarcinoma-intestine-pancreas/pancreatitis-associated protein
H3K27	Lysine 27 of histone H3
HRE	Hypoxia-response element
IH	Intermittent hypoxia
IL	Interleukin
KDMs	Lysine demethylases
MC4R	Melanocortin 4 receptor
miR	MicroRNA
NADPH	Nicotinamide adenine dinucleotide phosphate reduced form
Nox	NADPH oxidase(s)
NPY	Neuropeptide Y
NTS	Neurotensin
OSA	Obstructive sleep apnea
POMC	Proopiomelanocortin
PYY	Peptide YY
QRFP	Pyroglutamylated RFamide peptide
*Reg*	Regenerating gene
RETN	Resistin
ROS	Reactive oxygen species
SAS	Sleep apnea syndrome
siRNA	Small interfering RNA
TNF-α	Tumor necrosis factor-α
TSA	Trichostatin A
